# Outcomes and Prognostic Factors of Salvage Surgery for Recurrent Oral Squamous Cell Carcinoma

**DOI:** 10.3390/cancers18193113

**Published:** 2026-09-25

**Authors:** Pietro Orlando, Letizia Flammia, Giuseppe Licci, Luca Novelli, Saverio Caini, Angelo Cannavicci, Lapo Serni, Oreste Gallo

**Affiliations:** 1 Otorhinolaryngology Clinic, Careggi University Hospital, Florence—Largo Brambilla, 3, 50134 Florence, Italy; pietro.orlando@unifi.it (P.O.);; 2 Otorhinolaryngology Unit, Ospedale Civile di Vigevano, ASST Pavia, 27029 Vigevano, Italy; 3 Institute of Histopathology and Molecular Diagnosis, Careggi University Hospital, 50134 Florence, Italy; 4 Cancer Risk Factors and Lifestyle Epidemiology Unit, Institute for Cancer Research, Prevention, and Clinical Network (ISPRO), 50139 Florence, Italy; 5 Research Unit in Periodontology and Periodontal Medicine, Department of Experimental and Clinical Medicine, University of Florence, 50121 Florence, Italy

**Keywords:** recurrent oral squamous cell carcinoma, salvage surgery, surgical margin status, lymph node involvement, overall survival, disease-free survival

## Abstract

Background: Recurrent oral squamous cell carcinoma (rOSCC) is associated with a poor prognosis, and evidence supporting risk stratification in the salvage setting remains limited. The prognostic value of conventional staging systems and tumor-related characteristics after recurrence has not yet been fully established. Methods: We conducted a single-center observational study including patients with rOSCC who had previously undergone treatment for pT1–2 N0M0 OSCC, all subsequently surgically salvaged. Clinical, histopathological, and recurrence-related variables were collected. Recurrent tumors were classified according to the AJCC TNM staging system, the Composite Oral Cavity and Oropharynx Recurrence Staging System (CORSS), and the Same–Up–Down staging system (SUDss), a novel staging system developed by our group. Results: A total of 49 patients were included. Several pathological characteristics, including the rT3–4 category (*p* = 0.025), perineural invasion (*p* < 0.001), lymphovascular invasion (*p* = 0.003), greater depth of invasion (*p* = 0.011), nodal involvement (*p* = 0.002), and higher rTNM (*p* = 0.004) and CORSS stage (stage II *p* = 0.012; stage III *p* = 0.038) were associated with poorer OS in univariable analyses. In multivariable analysis, CORSS stage II (HR 2.89, 95% CI 1.04–8.04; *p* = 0.041) and stage III (HR 12.73, 95% CI 2.11–76.68; *p* = 0.005), as well as close/positive surgical margins (HR 2.57, 95% CI 0.99–6.71; *p* = 0.045) and pathological nodal involvement (HR 4.82, 95% CI 1.77–13.14; *p* = 0.002), were independently associated with poorer OS. Conclusions: CORSS appears to offer complementary prognostic value compared with conventional rTNM in patients with surgically treated rOSCC, although formal comparative validation of discrimination and calibration is warranted. Pathological lymph node involvement and close or positive surgical margins were the most relevant independent adverse prognostic factors. These findings identify higher-risk patients but do not, by themselves, establish a benefit from treatment intensification.

## Simple Summary

Oral squamous cell carcinoma (OSCC) frequently recurs even in patients with early-stage disease. When recurrence occurs, salvage surgery often represents the only potentially curative treatment; however, predicting patient outcomes remains challenging. In this study, we analyzed 49 consecutive patients with recurrent OSCC who underwent salvage surgery at our institution. Clinical, pathological, and recurrence-related factors were evaluated, and different staging systems were compared in terms of their ability to predict prognosis. Although several variables were associated with poorer survival in univariate analysis, only close or positive surgical margins and lymph node involvement were identified as independent adverse prognostic factors in multivariable analysis. Among the staging systems assessed, CORSS remained independently associated with OS and appeared to provide complementary prognostic information beyond rTNM, although formal comparative measures of discrimination and calibration were not performed.

## 1. Introduction

Oral squamous cell carcinoma (OSCC) is the ninth most common malignant tumor worldwide, with approximately 350,000 new diagnoses and more than 170,000 deaths annually [1]. The oral tongue is the most common subsite and is associated with the poorest prognosis, largely due to the high incidence of occult cervical lymph node metastasis [2]. Despite advances in multimodal treatment, up to 30–40% of patients experience disease recurrence within the first two years, even after early-stage tumors treated with curative intent. Recurrent OSCC (rOSCC) represents a distinct clinical challenge, with a markedly worse prognosis compared with primary disease and limited evidence to guide optimal management strategies, with reported 5-year overall survival (OS) rates ranging from 30% to 50% [3,4]. Salvage surgery remains the main curative option for rOSCC; however, outcomes are highly heterogeneous and difficult to predict using conventional staging systems. In particular, traditional TNM staging does not account for recurrence-related tumor biology, and its prognostic value in the recurrent setting remains limited. Similarly, although surgical margin status is a well-established prognostic factor in primary OSCC, its role and optimal threshold in rOSCC are still not clearly defined [5,6,7]. To address this gap, alternative recurrence-based staging systems, such as the Composite Oral Cavity and Oropharynx Recurrence Staging System (CORSS) and the Same–Up–Down staging system (SUDss), have been proposed, but their clinical utility remains controversial and insufficiently validated in homogeneous cohorts of surgically treated patients [8,9]. Moreover, the interaction between pathological tumor features, recurrence dynamics, and surgery in determining prognosis remains poorly understood. These systems capture different dimensions of recurrent disease: rTNM restages the recurrent tumor using conventional anatomic T/N/M categories, CORSS incorporates both the initial stage and the extent of recurrence, whereas SUDss reflects the direction of T-category change between the primary and recurrent tumors [8,9]. Their differing structure therefore provides complementary, rather than interchangeable, approaches to prognostic stratification.

Therefore, this study aimed to evaluate the prognostic impact of clinicopathological characteristics and recurrence-related factors, as well as the ability of the TNM, CORSS, and SUDss to stratify survival outcomes in patients undergoing salvage surgery for rOSCC.

## 2. Materials and Methods

We conducted a single-center observational study with prospective data collection, enrolling patients diagnosed with rOSCC who had previously undergone surgical treatment for pT1–2 N0M0 OSCC at our referral hospital in Florence, Italy, between January 2016 and December 2024. The restriction to initially early-stage, node-negative, nonmetastatic disease was intentional to obtain a more homogeneous baseline cohort and reduce heterogeneity related to prior surgical approach, neck management, reconstructive requirements, adjuvant treatment, and overall treatment burden, all of which differ substantially in patients initially treated for advanced OSCC and may influence salvage outcomes.

All patients were surgically salvaged. Before treatment, each patient with rOSCC was discussed by a multidisciplinary team comprising head and neck surgeons, pathologists, radiologists, plastic surgeons, and radiation oncologists. In our practice, lateral neck dissection is routinely performed in all patients with recurrent disease. However, omission of neck dissection may be considered in selected patients with a clinically and radiologically negative neck, particularly in the presence of a very small recurrent tumor and/or poor performance status.

The study was conducted in accordance with the Declaration of Helsinki. Informed consent was obtained from all participants. Ethical review and approval were waived because the study involved the analysis of de-identified patient data and did not include any interventions beyond standard clinical care (waiver/reference protocol no. 19648, dated 20 October 2021).

For each patient, demographic and clinical data were collected, including sex, age, and smoking and alcohol habits. For the primary OSCC, we recorded the oral cavity subsite (oral tongue, floor of mouth, retromolar trigone, cheek, gingiva, hard palate, and lip), treatment modality (surgery ± adjuvant therapy), tumor stage according to the American Joint Committee on Cancer (AJCC) TNM classification (8th edition), and interval between primary and recurrent tumor. For rOSCC, we also recorded the recurrence subsite and histopathological variables, including perineural invasion (PNI), lymphovascular invasion (LVI), depth of invasion (DOI), worst pattern of invasion (WPOI), and surgical margin status, as assessed by intraoperative frozen sections (IFSs) and final paraffin-embedded histopathology. A histopathological margin of 5 mm was adopted as the cutoff to define clear surgical margins, in accordance with the conventional criteria used for primary OSCC. Specifically, margins were classified according to the minimum distance between invasive carcinoma and the resection margin as involved or positive (<1 mm), close (1–5 mm), or clear or negative (>5 mm) [10]. Patients were subsequently staged according to three classification systems: the AJCC TNM staging system for recurrent disease (rTNM), the CORSS, and the SUDss. The rTNM is the currently adopted staging system for rOSCC and mirrors the classification used for primary tumors. The CORSS, developed in 1999, combines the initial TNM stage and the extent of recurrence (local, regional, or distant) through conjunctive consolidation, allowing patient stratification into four prognostic classes [8]. Finally, the SUDss was originally developed by our group to predict disease-specific survival (DSS) and overall survival (OS) in patients with recurrent early-stage glottic cancer and was subsequently evaluated as a prognostic tool in recurrent head and neck malignancies. According to this system, recurrent tumors are classified as “down” when the recurrent T category (rT) is lower than the primary T category, “same” when the two categories are identical, and “up” when the rT category is higher than the primary T category [9]. At salvage, the decision to perform neck dissection was individualized according to the clinical and radiological status of the neck, previous neck treatment, and the patient’s surgical history. Accordingly, pathological nodal positivity could include occult metastases identified only after neck dissection.

Disease-free interval (DFI) was defined as the time between treatment of the rOSCC and diagnosis of a second recurrence. Follow-up was calculated from the date of salvage surgery until death or the most recent clinical assessment.

Overall survival (OS) and DFI were defined as the primary endpoints. OS was selected as the principal survival endpoint; among the 31 deaths observed during follow-up, 26 (83.9%) were attributable to rOSCC progression, so OS closely approximated disease-specific mortality in this cohort while still capturing all-cause mortality.

Descriptive statistics were calculated, and data normality was assessed using the Shapiro–Wilk test. Associations between rOSCC and categorical variables were evaluated using the chi-squared test or Fisher’s exact test, as appropriate. Continuous variables were compared using the Student’s *t*-test for normally distributed data and the Mann–Whitney U test for non-normally distributed data.

OS was estimated using the Kaplan–Meier method. Univariable and multivariable Cox proportional hazards regression analyses were performed to identify independent prognostic factors for OS. The primary-to-recurrence interval was initially categorized into tertiles to preserve balanced subgroup sizes and was also evaluated as a continuous variable. The multivariable OS model was obtained by backward selection and was prespecified to retain no more than three variables, balancing clinical relevance against model stability in view of the 31 OS events (approximately 10 events per retained variable); all 31 deaths were included as events in the OS model. Post-salvage adjuvant treatment was not entered as an independent covariate because it was not randomly assigned but selected according to pathological risk factors, previous treatment, patient characteristics, and multidisciplinary evaluation, raising a substantial risk of confounding by indication; moreover, the small treatment subgroups would have yielded unstable estimates.

The statistical analyses were performed with SPSS software (IBM Corp. Release 2020, IBM SPSS Statistics for Macintosh, Version 27.0, Armonk, NY, USA). All statistical tests were two-sided, and *p*-values < 0.05 were considered statistically significant.

## 3. Results

During the study period, 137 patients with pT1–T2 N0M0 OSCC were treated at our institution. Of these, 53 developed recurrent rOSCC (38.7%); however, 4 of them had distant recurrence and were not amenable to salvage surgery; thus, only 49 patients were included in the present analysis. Demographic and clinical characteristics are displayed in Table 1.

The mean primary-to-recurrence interval was 43.1 ± 43.6 months. The median follow-up after salvage surgery was 60 months.

Among our 49 patients, recurrence was predominantly local, occurring in 40 patients (81.6%), while 9 patients (18.4%) developed clinical locoregional relapse.

The oral tongue emerged as the principal site of recurrence, accounting for 29 cases (59.2%), followed by the floor of the mouth in 7 cases (14.3%) and gingiva in 6 cases (12.2%).

All patients underwent salvage surgery, followed by adjuvant treatments including radiation therapy (RT), chemotherapy (CHT), or combined chemoradiotherapy (CHRT) in 18.4%, 6.1%, and 8.2% of cases, respectively. At the time of primary OSCC treatment, 28 patients (57.1%) had undergone neck dissection. Neck dissection was performed during salvage treatment in 18 out of 21 patients (85.7%), including selective neck dissection in 9 patients (18.4%) and modified radical neck dissection in 9 patients (18.4%).

Reconstruction of the surgical defect was required in 19 patients (38.8%). Among these, 10 patients (52.6%) underwent reconstruction with a regional rotational flap and 9 (47.4%) with a microvascular free flap.

Histopathological examination of the recurrent tumors demonstrated perineural invasion in 16 patients (32.7%), lymphovascular invasion in 9 (18.4%), and WPOI 4–5 in 12 (24.5%). Close or positive margins on final histopathological examination were observed in 18 patients (36.7%).

According to the rTNM staging system, pathological rOSCC stages were distributed as follows: rpT1 in 18 patients (36.7%), rpT2 in 16 (32.7%), rpT3 in 3 (6.1%), rpT4a in 11 (22.4%), and rpT4b in 1 (2.0%). Overall, 10 patients (20.4%) presented with pathological nodal involvement (rpN+). According to the AJCC stage grouping, 19 patients (38.8%) were classified as stage I, 10 (20.4%) as stage II, 7 (14.3%) as stage III, and 13 as stage IV (26.5%).

According to the CORSS classification, 20 patients (40.8%) were assigned to stage I, 18 (36.7%) to stage II, and 11 (22.5%) to stage III.

Finally, using the SUDss, the recurrent T category was higher than the primary T category (“up”) in 26 patients (53.1%), lower than the primary T category (“down”) in 7 patients (14.3%), and unchanged (“same”) in 16 patients (32.7%).

During follow-up, 20 of 49 patients (40.8%) developed a second recurrence, and 31 deaths occurred, 26 of which (83.9%) were directly attributable to rOSCC progression; the remaining 5 deaths were due to non-cancer-related causes or comorbidities. Median and mean OS were 50.0 (95% CI: 16.7–83.3) and 45.8 months (95% CI: 32.3–59.3), respectively. DFI between rOSCC and second recurrence was 20.8 months. The primary-to-recurrence interval was not significantly associated with OS when modeled as a continuous variable, consistent with the tertile-based analysis.

Univariable Cox regression identified several factors associated with worse OS in patients with rOSCC. These included the rT3–4 category (HR 2.91, 95% CI 1.14–7.40; *p* = 0.025), recurrent pathological nodal involvement (pN+) (HR 4.10, 95% CI 1.71–9.84; *p* = 0.002), rDOI > 5 mm (HR 11.36, 95% CI 1.46–88.2; *p* = 0.011), rPNI+ (HR 24.37, 95% CI 4.86–122.35; *p* < 0.001), and rLVI+ (HR 8.71, 95% CI 2.09–36.20; *p* = 0.003) (Figure 1). Concerning staging systems, both the rTNM (HR 2.25, 95% CI 1.03–4.93; *p* = 0.042) and CORSS classifications identified patients with stage II and III disease as having significantly worse OS than those with stage I disease (CORSS stage II: HR 3.20, 95% CI 1.29–7.89; *p* = 0.012; stage III: HR 5.31, 95% CI 1.10–25.74; *p* = 0.038). In contrast, the SUDss was not significantly associated with OS. In the three-category analysis using the “down” group as the reference, neither the “same” category (HR, 0.63; 95% CI, 0.19–2.05; *p* = 0.445) nor the “up” category (HR, 0.94; 95% CI, 0.33–2.70; *p* = 0.910) was significantly associated with survival. Consistently, when patients classified as “up” were compared with those in the combined “down/same” group, no statistically significant difference in OS was observed (log-rank *p* = 0.254) (Figure 2).

In multivariable analysis, only the CORSS classification retained independent prognostic significance among the staging systems, with stage II (HR 2.89, 95% CI 1.04–8.04; *p* = 0.041) and stage III disease (HR 12.73, 95% CI 2.11–76.68; *p* = 0.005) independently predicting worse OS. In addition, close or positive surgical margins (HR 2.57, 95% CI 0.99–6.71; *p* = 0.045) and pathological nodal involvement (HR 4.82, 95% CI 1.77–13.14; *p* = 0.002) remained independently associated with reduced survival. The final OS model therefore included three covariates and all 31 observed deaths as events.

At univariable analysis, none of the clinical, histopathological, or staging-related variables significantly influenced DFI, although close or positive surgical margins showed a nonsignificant trend toward worse outcomes. After multivariable adjustment, no independent predictors of DFI emerged.

Results of univariate and multivariate analyses are summarized in Table 2 and Table 3, respectively.

## 4. Discussion

OSCC remains one of the most frequent and clinically challenging malignancies of the head and neck, largely due to the complex anatomy of the oral cavity and its impact on function and quality of life. Although early-stage disease is generally amenable to curative-intent surgery, recurrence remains a clinically relevant concern, underscoring the importance of close surveillance and improved risk-stratification strategies [3,11,12,13,14].

In our cohort, 49 of 137 consecutive patients initially treated for T1-2 OSCC developed a locoregional recurrence. The oral tongue was the most common site of recurrence, followed by the floor of the mouth and gingiva. Recurrences involving these subsites may exhibit more aggressive behavior because of their rich vascular supply, proximity to the bone, and anatomical constraints that might limit the achievement of adequate surgical margins. The deliberate restriction to patients with initially pT1–pT2 N0M0 disease reduced heterogeneity related to prior treatment exposure and baseline tumor burden, but it also limits the generalizability of our findings to recurrences arising after initially advanced OSCC.

In univariable analysis, higher rpT category, rpN+, rPNI+, rLVI+, and rDOI > 5 mm were associated with poorer OS, consistent with the existing literature [3,4,15,16,17,18,19,20,21,22,23,24,25,26,27,28,29,30,31,32,33]. Conversely, unlike prior studies, the primary-to-recurrence interval did not show a significant impact on survival in our cohort [4,5,16,26,27,28]. Several factors may account for this finding. A substantial proportion of patients who subsequently developed recurrent disease had close or positive surgical margins after resection of the primary OSCC. Therefore, some lesions classified as recurrences may have arisen from persistent microscopic disease, whereas others may have represented metachronous second primary tumors or second-field tumors developing within genetically altered mucosa and not necessarily clonally related to the index OSCC. Indeed, subsequent oral cavity tumors may represent true local recurrences, second primary tumors, or second-field tumors, each characterized by a potentially different biological origin [34]. Molecular evidence supports this biological distinction. In paired head and neck squamous cell carcinoma specimens obtained before and after radiotherapy, Deganello et al. used TP53 and microsatellite markers to distinguish concordant true recurrences from genetically discordant second primary malignancies and described partially related lesions consistent with a second-field process [35]. Although that study concerned HNSCC after radiotherapy rather than exclusively recurrent oral cavity tumors, it illustrates why clinical criteria alone cannot reliably establish clonality. Because molecular or clonality analyses were unavailable in our cohort, true recurrence, second primary tumors, and second-field tumors could not be analyzed separately, which may have attenuated the association between recurrence timing and outcome. In addition, although tertile categorization was chosen to maintain balanced subgroup sizes, the primary-to-recurrence interval remained nonsignificant when modeled continuously; the limited sample size precluded stable assessment of more complex nonlinear relationships, such as restricted cubic splines.

In multivariable analysis, close or positive surgical margins and rpN+ status emerged as independent predictors of poorer OS. In particular, close or positive margins were associated with worse survival, in agreement with previous studies [4,7,17,27,36,37,38,39,40,41,42]. In our cohort, the conventional 5 mm threshold used to define clear margins in primary OSCC retained prognostic relevance in the recurrent setting, as patients with margins < 5 mm experienced poorer OS; however, these data do not establish a universal oncologically safe cutoff for all salvage settings. Surgical margin status was not significantly associated with post-salvage DFI. This apparent discrepancy may reflect differences between the two endpoints rather than a true lack of an effect on disease control: death before documentation of a second recurrence may act as a competing event, while postoperative adjuvant treatment and the limited number of recurrence events may reduce statistical power. Moreover, although OS includes all-cause mortality, 26 of the 31 recorded deaths (83.9%) were directly attributable to rOSCC progression, indicating that OS closely tracked oncological outcome in this cohort. Nevertheless, associations of margin status or nodal involvement with OS should not be interpreted as direct proof of cancer-specific disease control.

In this cohort, achieving a histopathological margin > 5 mm was associated with more favorable OS and may represent a reasonable surgical objective during salvage treatment whenever oncologically and functionally feasible [12,36,37,38,43]. However, our data do not establish a universal 5 mm oncological safety threshold in recurrent surgical fields. Obtaining adequate margins is considerably more challenging in rOSCC than in primary tumors: previous ablative surgery may result in tissue loss, fibrosis, scarring, distortion of normal anatomical planes, and reduced availability of healthy surrounding tissue, while the extent of resection must be balanced against preservation of speech, swallowing, mastication, and airway function. Margin assessment may also be particularly demanding for the pathologist because of treatment-related tissue alterations, specimen distortion and shrinkage, difficulties in orientation, and the possible presence of small or dispersed peripheral tumor nests. Beyond anatomic margin distance, molecular proliferative pathways may contribute to OSCC aggressiveness. Cheng et al., using OSCC cell lines and in vivo tumorigenesis models, showed that NFκB inhibition suppressed cell proliferation, promoted apoptosis, and reduced tumorigenic behavior [44]. Although not a study of salvage surgical margins, these findings reinforce the broader concept that anatomic margin distance alone cannot capture the full biological aggressiveness of recurrent OSCC.

The presence of rpN+ also independently predicted worse survival, highlighting the adverse prognostic impact of regional disease in the salvage setting. This finding is consistent with previous evidence showing that nodal involvement at recurrence represents a major determinant of outcome after salvage surgery [25,28,29,45]. Importantly, the prognostic associations of rpN+ and close/positive margins should not be interpreted as evidence that these patients necessarily benefit from treatment intensification, because the present study was not designed to compare therapeutic strategies or treatment response. In ovarian cancer, Wang et al. reviewed how the tumor immune microenvironment can modulate the efficacy of immunotherapy [46]; although this evidence derives from a different disease site and should not be extrapolated directly to rOSCC, it illustrates the broader principle that therapeutic response depends on biological context beyond anatomic risk factors. Prospective rOSCC-specific studies are required before treatment escalation can be recommended.

Although PNI, LVI, and DOI showed strong associations with OS in univariable analyses, they were not retained as independent predictors in the final multivariable model. Their exclusion primarily reflects uneven event distribution across subgroups, the small sample size, and the already wide confidence intervals observed in univariable analyses, together with the backward model-selection strategy; it should not be interpreted as evidence of biological irrelevance. Gong et al. identified a cuproptosis-related lncRNA signature in OSCC associated with survival, tumor-microenvironment characteristics, and predicted immunotherapy response [47]. Ramalingam et al., in their study on KRAS-associated colorectal cancer, used bioinformatics and machine learning approaches to investigate lncRNA/miRNA/mRNA axes and screen potential miRNA inhibitors [48]. Although the latter study concerns a different tumor site, these findings illustrate how molecular and computational biomarkers may complement conventional clinicopathological staging. Additional evidence from other head and neck and squamous carcinoma contexts also supports the potential relevance of biologically driven markers: Huang et al. used a network-pharmacology approach to investigate molecular mechanisms in nasopharyngeal carcinoma [49], whereas Luo et al. linked PSD3 to autophagy-associated MHC-I immune modulation in esophageal squamous cell carcinoma [50]. Because these studies concern different disease sites, their findings should not be extrapolated directly to rOSCC; rather, they illustrate the potential value of integrating molecular and immune parameters into future risk models.

With regard to staging systems, both rTNM and CORSS were significantly associated with OS in univariable analysis. However, only CORSS retained independent prognostic significance after multivariable adjustment. This finding suggests that a recurrence-specific classification incorporating characteristics of both the primary and recurrent tumors may offer complementary prognostic information, but statistical significance in a multivariable model does not by itself establish superior discrimination or calibration compared with rTNM. The prognostic performance of rTNM may be limited in the recurrent setting by the heterogeneity of disease patterns included within the same stage groups; stages I and II encompass exclusively local disease, whereas stage IV includes markedly different clinical conditions, ranging from locally advanced T4N0M0 tumors to distant metastatic disease. Assignment of the recurrent T category may also be challenging in multifocal local disease or exclusively nodal or distant recurrence. Similarly, the SUDss did not significantly stratify OS in our cohort. Neither the three-category comparison nor the dichotomized analysis comparing patients classified as “up” with those classified as “same/down” showed significant survival differences (log-rank *p* = 0.254). This limited performance may partly reflect the inclusion of only patients initially treated for pT1–pT2 OSCC, which restricted the range of possible T-stage changes, and the fact that SUDss does not account for nodal involvement, surgical margin status, or other adverse histopathological features. More quantitative prediction approaches may ultimately complement conventional categorical staging. In cervical cancer, Ye et al. combined deep convolutional features extracted from H&E whole-slide images with a random forest model to predict recurrence and metastasis risk [51]. Although this work concerns a different disease site, it illustrates the potential of computational prediction models that would require dedicated validation in recurrent OSCC.

CORSS should therefore not be interpreted as a stand-alone determinant of individual treatment. Salvage decisions should integrate tumor-related risk with performance status, comorbidities, nutritional status, previous treatments, expected surgical morbidity, functional outcomes, and postoperative quality of life. As a case-based example outside OSCC, Huang et al. described severe systemic immune-related neurological and hematological conditions associated with tumors or infection [52]. Although this report does not provide evidence for rOSCC management, it illustrates the broader clinical complexity that may influence treatment tolerance and decision-making beyond tumor staging alone.

Beyond conventional anatomical and pathological variables, future prognostic models may benefit from integrating molecular and immune characteristics. In OSCC, Gong et al. identified a cuproptosis-related lncRNA signature associated with survival, tumor-microenvironment characteristics, and predicted immunotherapy response [49]. Huang et al., using network pharmacology in nasopharyngeal carcinoma, identified candidate molecular targets and cancer-related pathways and also explored prognostic signals in head and neck squamous cell carcinoma datasets [53]. Luo et al., in their study on esophageal squamous cell carcinoma, linked PSD3-associated autophagic flux to MHC-I expression and immune-cell infiltration [54]. The latter studies concern different anatomical sites and should not be interpreted as direct validation in recurrent OSCC; rather, they support the broader rationale for integrating molecular and immune biology with clinicopathological risk models.

Taken together, these observations indicate that clinicopathological staging and margin-based risk assessment should be interpreted within the broader biological and patient-specific context rather than as isolated determinants of prognosis or treatment.

Overall, our findings suggest that CORSS may offer complementary prognostic value compared with rTNM alone by integrating features of both the primary and recurrent disease. However, statistical significance does not inherently prove superior predictive discrimination or calibration. The CORSS stage III estimate should be interpreted particularly cautiously because it was based on only 11 patients and was accompanied by a wide confidence interval, indicating substantial uncertainty around the precise effect size. Before CORSS can influence individual treatment escalation or de-escalation in the salvage setting, external validation in larger, independent, preferably multicenter cohorts is required, together with formal assessment of discrimination and calibration using measures such as Harrell’s C-index and time-dependent AUC.

This study has several limitations, including its single-center design, relatively small sample size, and potential selection bias toward patients considered eligible for salvage surgery. The inclusion of only patients previously treated for pT1–pT2 N0M0 OSCC may limit generalizability to recurrences arising after more advanced primary disease and may have reduced the discriminatory ability of the staging systems evaluated, particularly SUDss. Although the final OS model retained only three variables for 31 deaths, consistent with an approximate 8–10 events-per-variable framework, the limited and uneven distribution of events across subgroups produced wide confidence intervals and may explain why PNI, LVI, and DOI were not retained after multivariable adjustment. The CORSS stage III subgroup was small (n = 11), further limiting precision. In addition, categorization of continuous variables such as the primary-to-recurrence interval into tertiles, used to preserve balanced subgroup sizes, may have masked nonlinear prognostic associations that more complex modeling or spline analyses could detect in larger cohorts. No molecular or clonality analyses were available to distinguish true recurrence from second primary or second-field tumors. Post-salvage adjuvant treatment was heterogeneous and non-randomly assigned according to adverse pathological features and prior treatment, so residual confounding by indication cannot be excluded. Finally, variability in follow-up duration and the limited number of second-recurrence events may have affected the detection of post-salvage recurrence.

Larger prospective multicenter studies are needed to externally validate CORSS with formal assessment of discrimination and calibration, to determine whether the conventional 5 mm margin threshold can be reliably generalized to recurrent OSCC, and to evaluate treatment strategies prospectively rather than infer therapeutic benefit from prognostic associations. Future risk models should integrate recurrence-specific staging with clinical, histopathological, molecular, and immune-related characteristics. Such integration is supported by emerging evidence from OSCC and from other squamous/head and neck contexts, while disease-site-specific validation remains essential.

## 5. Conclusions

rOSCC represents a biologically aggressive and heterogeneous disease. In this selected cohort of patients undergoing salvage surgery, pathological nodal involvement and close or positive surgical margins were independently associated with reduced survival. Obtaining an adequately negative margin remains an important surgical objective whenever oncologically and functionally feasible, but our findings do not establish a universal 5 mm oncological safety threshold in recurrent disease. CORSS provided complementary prognostic information beyond conventional rTNM, but its apparent value should not be interpreted as proven superior discrimination and requires external validation in larger cohorts. Finally, N+ and/or R+ status should be regarded as markers of increased prognostic risk rather than as direct evidence that treatment intensification improves outcome.

## Figures and Tables

**Figure 1:**
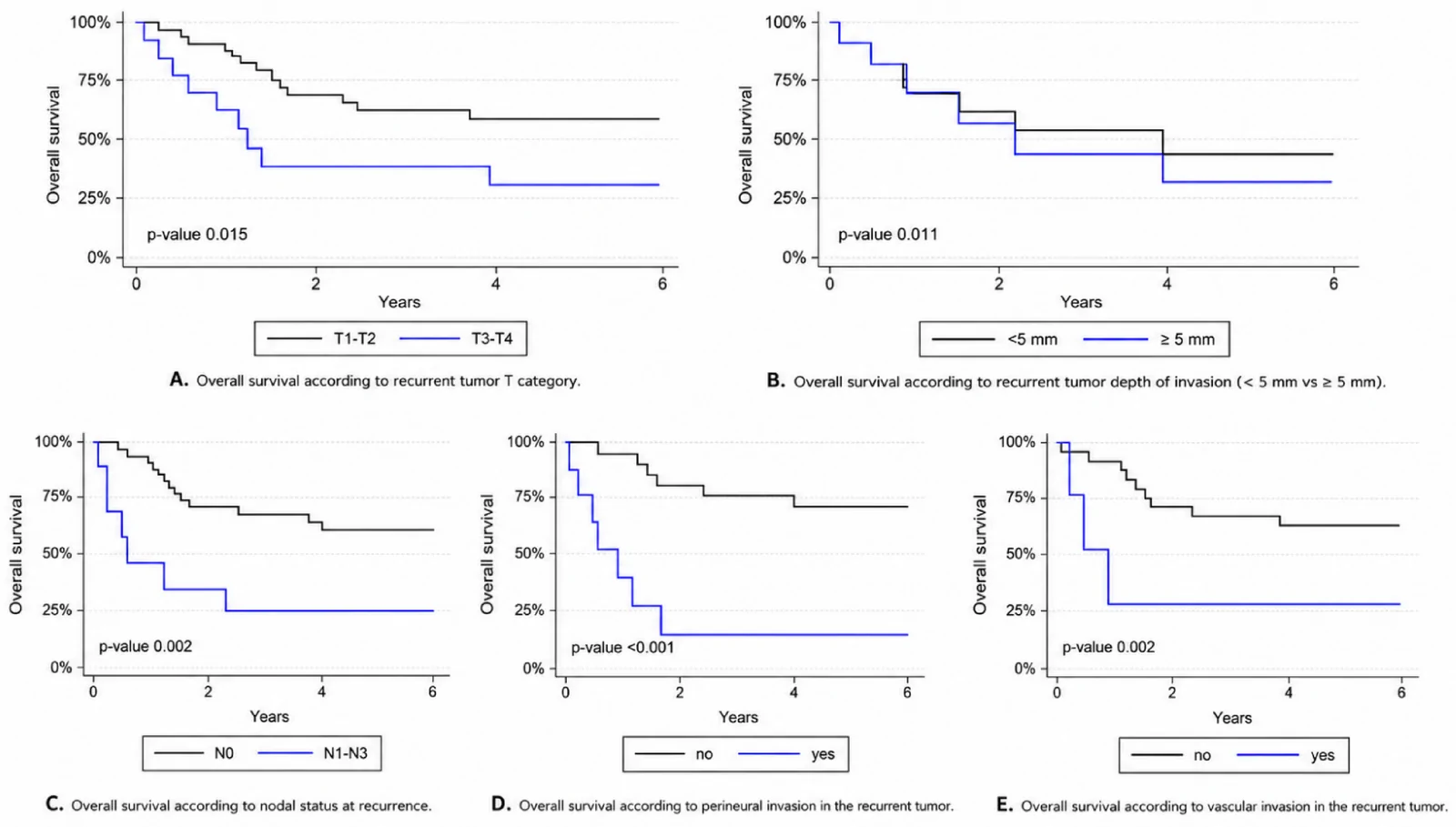
Kaplan–Meier survival curves stratified according to recurrent T category, recurrent depth of invasion, recurrent cervical pathological nodal status, recurrent perineural invasion, and recurrent lymphovascular invasion.

**Figure 2:**
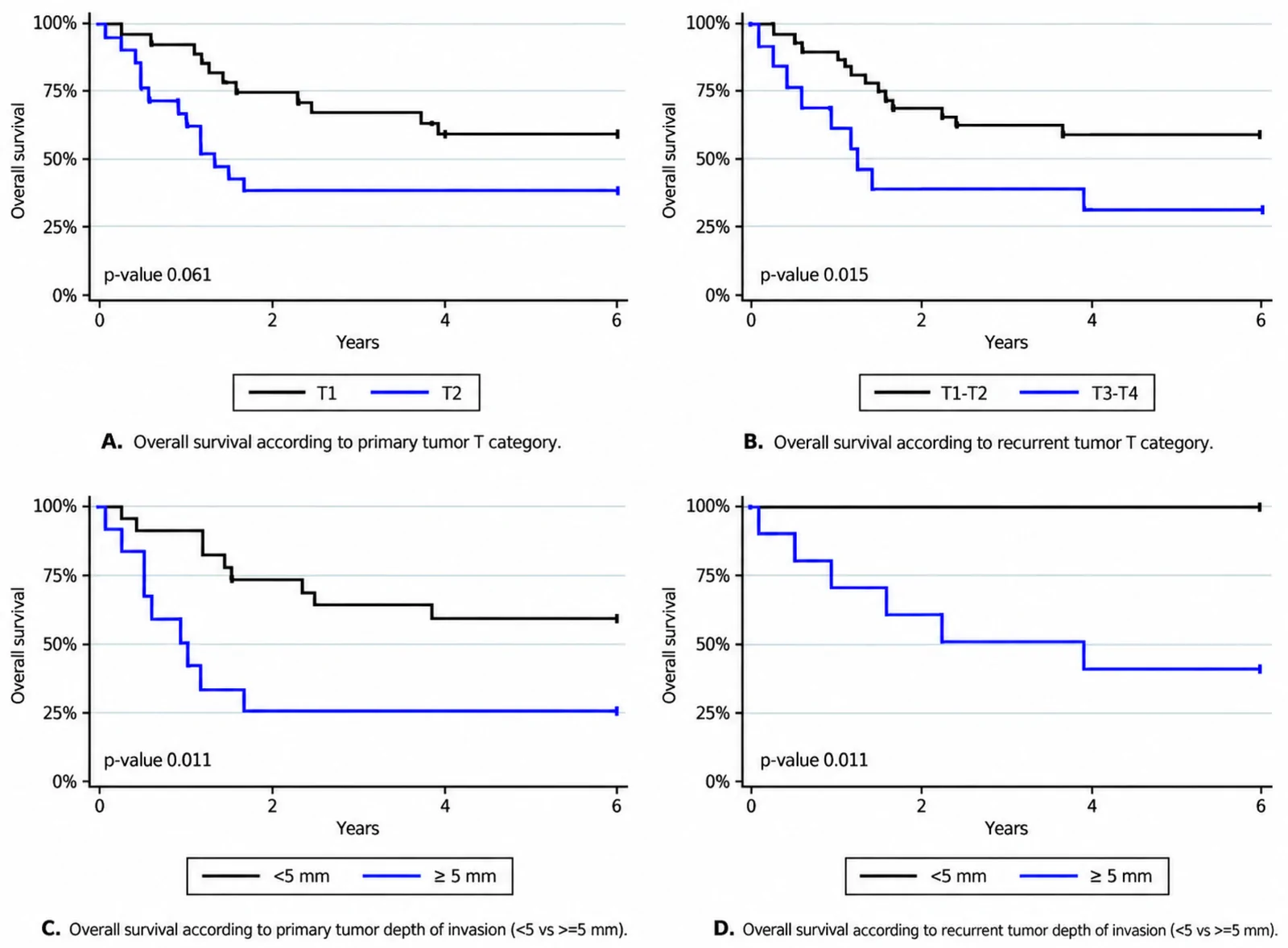
Kaplan–Meier survival curves stratified according to rTNM, CORSS, and SUD stage groups. CORSS: Composite Oral Cavity and Oropharynx Recurrence Staging System; SUD: Same–Up–Down.

**Table 1:** Clinical, pathological, and treatment characteristics of recurrent oral squamous cell carcinoma.

Characteristic	Category/Statistic	N	%
Demogtraphic and Clinical Characteristics			
Sex	Male	18	36.7
	Female	31	63.3
Alcohol use	No	42	85.7
	Yes	7	14.3
Smoking status	Current smoker	12	24.5
	Never smoker	29	59.2
	Former smoker	8	16.3
Primary-to-recurrence interval	Mean (SD), months	43.1 (43.6)	-
Follow-up after salvage surgery	Median, months	60.0	-
Primary OSCC Treatment			
Neck dissection at primary treatment	No	21	42.9
	Yes	28	57.1
rOSCC and Salvage Treatmente			
Pattern of recurrence	Local	40	81.6
	Locoregional	9	18.4
Anatomic subsite of rOSCC	Tongue	29	59.2
	Floor of the mouth	7	14.3
	Buccal mucosa	2	4.1
	Retromolar trigone	2	4.1
	Palate	2	4.1
	Gingiva	6	12.2
	Lip	1	2.0
Treatment of recurrence	Salvage surgery alone	33	67.3
	Salvage surgery + RT	9	18.4
	Salvage surgery + CHT	3	6.1
	Salvage surgery + CHRT	4	8.2
Neck dissection at salvage	None	31	63.3
	SND	9	18.4
	mRND	9	18.4
Reconstruction	No	30	61.2
	Yes	19	38.8
Reconstruction type (among reconstructed patients) *	Regional rotational flap	10	52.6
	Microvascular free flap	9	47.4
Pathological and Staging Characteristics of rOSCC			
rpT category	T1	18	36.7
	T2	16	32.7
	T3	3	6.1
	T4a	11	22.4
	T4b	1	2.0
rpN category	N0	39	79.6
	N1	6	12.2
	N2	3	6.1
	N3	1	2.0
rTNM stage (AJCC 8th edition)	I	19	38.8
	II	10	20.4
	III	7	14.3
	IV	13	26.5
CORSS stage	I	20	40.8
	II	18	36.7
	III	11	22.5
SUDss category	Down	7	14.3
	Same	16	32.7
	Up	26	53.1
Final surgical margin status	Clear/negative (>5 mm)	31	63.3
	Close/positive (≤5 mm)	18	36.7
DOI	≤5 mm	28	57.1
	>5 mm	21	42.9
Lymphovascular invasion	No	40	81.6
	Yes	9	18.4
Perineural invasion	No	33	67.3
	Yes	16	32.7
Worst pattern of invasion	WPOI 1–3	37	75.5
	WPOI 4–5	12	24.5
Post-salvage Outcomes			
Second recurrence	No	29	59.2
	Yes	20	40.8
Site of second recurrence (among patients with second recurrence) **	Local	11	55.0
	Regional	7	35.0
	Distant	2	10.0

* Percentages calculated among the 19 patients who underwent reconstruction. ** Percentages calculated among the 20 patients who developed a second recurrence. OSCC: oral squamous cell carcinoma; rOSCC: recurrent oral squamous cell carcinoma; RT: radiotherapy; CHT: chemotherapy; CHRT: chemoradiotherapy; DOI: depth of invasion; CORSS: Composite Oral Cavity and Oropharynx Recurrence Staging System; SUDss: Same–Up–Down staging system.

**Table 2:** Univariable Cox proportional hazards analyses of post-salvage disease-free interval and overall survival.

Variable/Category		Score	Post-Salvage DFI		Overall Survival	
Variable	Category	Score	HR (95% CI)	*p* Value	HR (95% CI)	*p* Value
Sex	Male	0	1.00		1.00	
	Female	1	1.24 (0.50–3.08)	0.650	1.16 (0.55–2.43)	0.694
Smoking status	Never	0	1.00		1.00	
	Former	1	0.62 (0.19–1.98)	0.418	2.05 (0.87–4.79)	0.099
	Current	2	0.93 (0.34–2.55)	0.892	0.58 (0.21–1.59)	0.292
Alcohol consumption	No	0	1.00		1.00	
	Yes	1	3.70 (0.72–19.08)	0.118	1.95 (0.76–4.98)	0.164
Primary OSCC T category	pT1	0	1.00		1.00	
	pT2	1	0.61 (0.26–1.46)	0.266	2.57 (1.23–5.37)	0.012
Primary OSCC–rOSCC interval	1st tertile	0	1.00		1.00	
	2nd tertile	1	0.21 (0.06–0.77)	0.019	1.78 (0.72–4.40)	0.208
	3rd tertile	2	0.31 (0.08–1.19)	0.088	1.59 (0.67–3.77)	0.293
Age at rOSCC diagnosis	1st tertile	0	1.00		1.00	
	2nd tertile	1	1.89 (0.70–5.14)	0.211	0.40 (0.14–1.11)	0.080
	3rd tertile	2	1.61 (0.53–4.91)	0.405	1.60 (0.70–3.65)	0.268
Recurrent T category	rT1	0	1.00		1.00	
	rT2	1	2.44 (0.85–6.98)	0.096	1.46 (0.56–3.80)	0.440
	rT3–4	2	1.13 (0.34–3.82)	0.839	2.91 (1.14–7.40)	0.025
Pathological nodal status at recurrence	rpN0	0	1.00		1.00	
	rpN+	1	0.89 (0.24–3.22)	0.855	4.10 (1.71–9.84)	0.002
Recurrent TNM stage	I–II	0	1.00		1.00	
	III–IV	1	1.76 (0.74–4.18)	0.0199	2.25 (1.03–4.93)	0.042
Recurrent depth of invasion	<5 mm	0	1.00		1.00	
	≥5 mm	1	2.35 (0.78–7.13)	0.131	11.36 (1.46–88.20)	0.011
Recurrent lymphovascular invasion	Absent	0	1.00		1.00	
	Present	1	1.02 (0.12–8.33)	0.988	8.71 (2.09–36.20)	0.003
Recurrent perineural invasion	Absent	0	1.00		1.00	
	Present	1	0.79 (0.21–3.01)	0.727	24.37 (4.86–122.35)	<0.001
Surgical margin status	Clear (≥5 mm)	0	1.00		1.00	
	Close/positive (<5 mm)	1	0.71 (0.26–1.93)	0.505	1.95 (0.86–4.43)	0.112
CORSS stage	I	0	1.00		1.00	
	II	1	0.93 (0.37–2.39)	0.887	3.20 (1.29–7.89)	0.012
	III	2	1.94 (0.23–16.42)	0.542	5.31 (1.10–25.74)	0.038
SUDss	Down	0	1.00		1.00	
	Same	1	2.50 (0.70–8.99)	0.160	0.63 (0.19–2.05)	0.445
	Up	2	2.61 (0.76–8.93)	0.125	0.94 (0.33–2.70)	0.910

OSCC: oral squamous cell carcinoma; rOSCC: recurrent oral squamous cell carcinoma; CORSS: Composite Oral Cavity and Oropharynx Recurrence Staging System; SUDss: Same–Up–Down staging system.

**Table 3:** Multivariable Cox proportional hazards analyses of post-salvage disease-free interval and overall survival.

Variable/Category		Score	Post-Salvage DFI		Overall Survival	
Variable	Category	Score	HR (95% CI)	*p* Value	HR (95% CI)	*p* Value
CORSS stage	I	0	—	—	1.00	
	II	1	—	—	2.89 (1.04–8.04)	0.041
	III	2	—	—	12.73 (2.11–76.68)	0.005
Final surgical margin status	Clear (≥5 mm)	0	—	—	1.00	
	Close/positive (<5 mm)	1	—	—	2.57 (0.99–6.71)	0.045
Pathological nodal status at recurrence	rpN0	0	—	—	1.00	
	rpN+	1	—	—	4.82 (1.77–13.14)	0.002

## Data Availability

Data supporting the results are available from the corresponding author upon reasonable request.
